# Supplementary figures and images for: Aesthetic judgment of architecture for Chinese observers
Source: PLoS One. 2022 Apr 5;17(4):e0265412. doi: 10.1371/journal.pone.0265412 (PMC8982842; doi:10.1371/journal.pone.0265412)

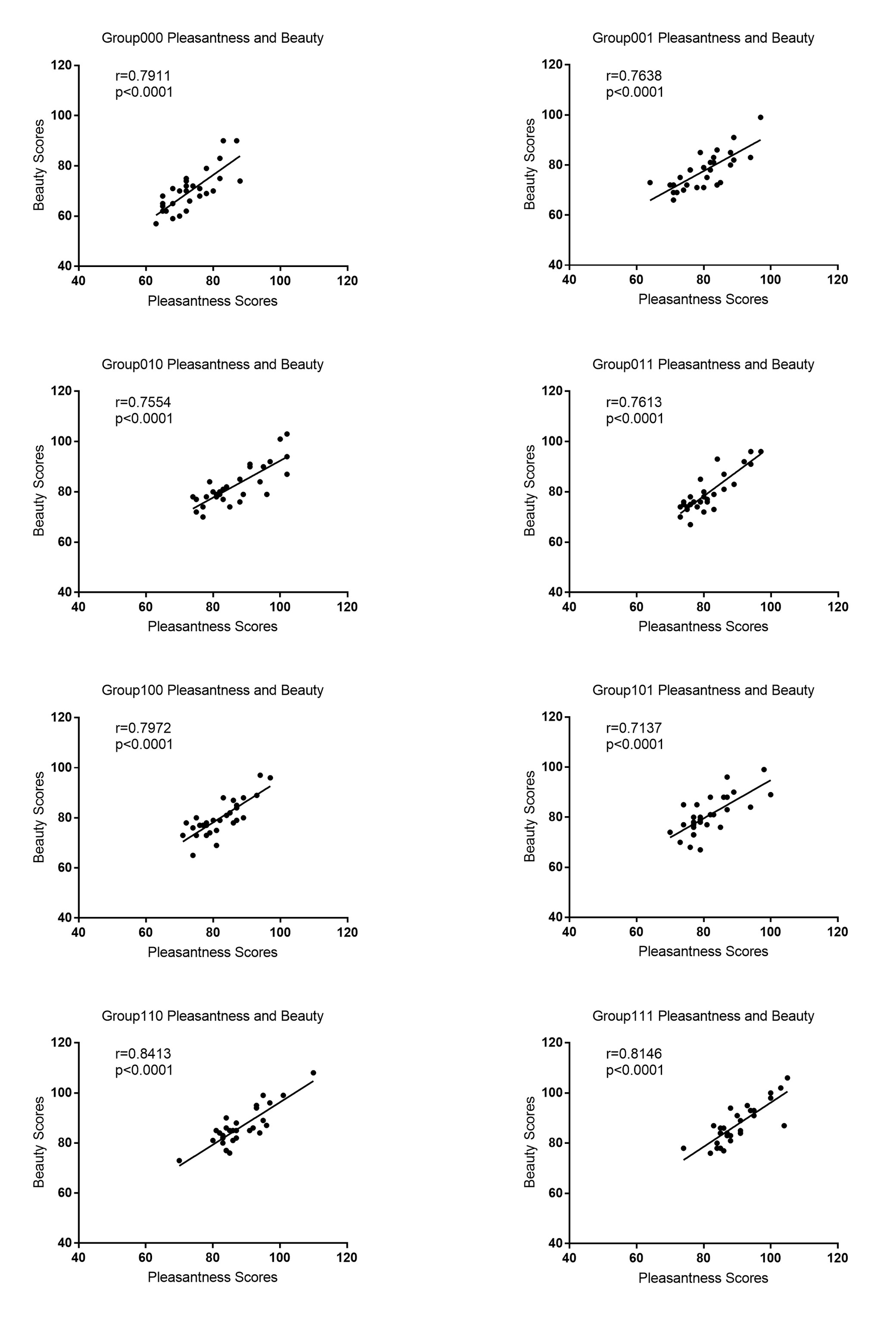

Supplement: S1 Fig — (TIF) [file pone.0265412.s001.tif]
