# Supplementary material for: Aesthetic judgment of architecture for Chinese observers
Source: PLoS One. 2022 Apr 5;17(4):e0265412. doi: 10.1371/journal.pone.0265412 (PMC8982842; doi:10.1371/journal.pone.0265412)
Supplement: Attachment — Submitted filename: Response to Reviewers.docx [file pone.0265412.s004.docx]

We would like to thank the editor and reviewers for their constructive suggestions. Please see below for our point-to-point replies.

SUMMARY:
There is abundant evidence of the aesthetic impact of differences in the design of architectural interiors. While ceiling height, openness, and contour differences have been analyzed in numerous Western studies on human aesthetic and pleasure scores, almost no Asian populations were used as experimental participants. In this experiment, the authors used the same picture stimuli and process settings used in many studies and replaced the participants with Chinese participants in the experiment, hoping to demonstrate that people growing up in different cultures have differences in aesthetics.

REVISIONS FOR THIS PAPER:

Reviewer #1: The paper investigated whether Chinese participants prefer architectural spaces characterised by features, such as curvilinear contours, high ceilings and open spaces. The results showed that Chinese participants prefer buildings with high ceilings, open spaces and curvilinear contours. While the paper seeks to answer an interesting question, I believe it is lacking in theoretical and methodological details which prevents the findings from being clear and convincing. I outline these limitations below.

Introduction:
1)The introduction is lacking important detail and makes some unsupported claims.

For example, on page 1, lines 4-5, the authors discuss the impact of environmental characteristics on ‘neurological and physiological response in humans’, however, there are no references cited for the claims made.

Thank you for pointing this out. We have added citations for this claim.

*“Environmental characteristics can trigger neurological and physiological responses in humans, thereby exerting a positive or negative impact on them [1-3]”.*

Also, on page 1, lines 16-17, the reference cited (4) is not appropriate here. 'Vers une architecture' by Le Corbusier is a manifesto (a collection of essays) for a new architectural style based on function and not empirical studies.

We have removed the reference.

Page 2 – first paragraph - the authors should provide more detail when summarising previous studies about the link between curvature, high ceilings and open spaces and aesthetic preference.

In this sense, the authors might consider including newer papers as well, such as

Coburn, A., Vartanian, O., Kenett, Y. N., Nadal, M., Hartung, F., Hayn-Leichsenring, G., … Chatterjee, A. (2020). Psychological and neural responses to architectural interiors. Cortex, 126, 217–241. https://doi.org/10.1016/j.cortex.2020.01.009

pre-print - Skov, M., Vartanian, O., Navarrete, G., Modroño, C., Chatterjee, A., Leder, H., … Nadal, M. (2021). Grey Matter Volume and architecture. Differences in Regional Grey Matter Volume Predict the Extent to which Openness influences Judgments of Beauty and Pleasantness of Interior Architectural Spaces. <https://doi.org/10.1101/2021.03.31.437827>

Thank you for pointing this out. We have carefully checked the *Introduction* and only kept claims that are well supported by the literature. Citations were added for these claims.

2)The authors should provide a clearer summary of the hypotheses and predictions at the end of the Introduction.

We have clearly stated the hypotheses and predictions:

*“Here, we investigate the architectural aesthetic preference of Chinese observers using the same set of pictures used in a previous study that investigated Western observers [8]. Since sensory experience, aesthetic preference, and culture significantly differ between Chinese and Western observers [19], we investigate whether Chinese observers show preference to architectural features that is similar to Western observers. In other words, we investigate whether Chinese observers also prefer curvilinear contours, high ceilings, and open space preference. If Chinese observers do not show preference to some of these features, it suggests that aesthetic judgment of that feature is experience or culture dependent. Otherwise, it suggests that preference to such features is potentially innate.*

*.”*

Method:
3)Please use research “participants” when referring to individuals who take part in psychological research, as it can be argued, it is more respectful of research volunteers.

We have now consistently used the word “participants”.

4)Please state more clearly how many participants completed this study.

5)Were any participants excluded from the analyses? If yes, please mention the exclusion criteria.

We initially planned to collect eye tracking data during the experiment but it turned out that the none of the initial 5 participants finished the experiment. Therefore, we abolished the plan to record eye tracking data and recruited 29 participants for the behavioural experiment. All the 29 participants were included in the analysis. We realized that it was confusing to say that 34 participants participanted in the experiment and only 29 was analyzed. Now, we no longer mention the 5 participant for the failed eye tracking experiment.

“*A total of 29 participants were included in this study, including 19 males (age: 23.05±1.99 years) and 10 females (age: 23.00±2.00 years). Among the subjects, 9 were undergraduates, 13 were master’s students, and 7 were PhD students.*”

6)The authors should offer a justification for the sample size used in this study.

The current study followed the same procedure used in the study by Vartanian.et al, and used the same stimuli. The study by Vartanian.et al recruited 18 participants and we recruited 29 participants. We have now mentioned that in *Discussion*. A more recent study using the same stimuli also only recruited 18 participants [1].

*“the study by Vartanian.et al. 2013 [8], and used the same stimuli. Twenty-nine participants were recruited in this study.”*

7)For more clarity, it would be useful if the authors could provide a subsection "Stimuli and Procedure"

We have now provided a new subsection *Stimuli and Procedure*.

8)It would be useful if the authors could provide a graphical illustration of an experimental trial for a better understanding of the research procedure.

We have added a new figure to the manuscript to illustrate the procedure (Fig. 2).

9)The authors should provide clearer details about the experimental procedure and the duration of the experiment. I find it confusing that the experiment length varied between 35 and 75 minutes. What is the justification for that?

Since the time to judge pleasantness and beauty was not limited, fast responders and slow responders took different amount of time. We have now explained this:

*“The time to make pleasantness and beauty judgement was self controlled and the total duration of the experiment was between 35 and 75 minutes.”*

Results:

10)It would be helpful to report the descriptive statistics - mean and SD for each condition.

We have now shown the mean and SD for each condition in a new table (Table 2).

11)It would be useful to mention whether the assumptions for ANOVA test have been met.

For repeated measures ANOVA, at least 4 assumptions should be met: normality at each category, no outlier, homogeneity of variance and sphericity. The violin plots show that data in each group are roughly normal and does not include outliers. There are no between-subjects factors in this study, so there is no need to do the Levene's test for Homogeneity of Variance. There are no factors have more than 2 levels, so no need to do the sphericity test. In conclusion, the assumptions for ANOVA test in this study have been met.

12)The results figures are lacking important detail. It would be helpful if individual data were plotted out. Violin plots would be more useful to see the spread and direction of the data. Also, they may help in identifying whether the data were normally distributed.

Please provide error bars 95% CI for all the figures.

We have changed all plots to violin plots and added the 95% CI.

13)It is very confusing why the authors have used the R results output tables instead of reporting all the F statements for main effects and interactions. It would be useful to report all the F statements in the results section.

Thank you for pointing it out. We have revised it.

14)Also, the results for the post hoc comparisons should be reported in the results section.

The description in the abstract is inaccurate and has been revised because all factors in this paper are at two levels, so there is no need for "post hoc comparisons". The interaction of the model in the paper is statistically significant, so a simple-simple effect test was performed after the ANOVA.

15)A table containing the mean differences across conditions would be helpful.

We have now shown the mean and SD for each condition in a new table (Table 2).

16)Please provide your data and analysis pipeline online if possible.

All data and analysis pipeline will be made online after the manuscript is accepted.

Discussion:

17)Please interpret your results in accordance with the main key hypotheses.

We have added discussions relating the results with the hypotheses.

*“The preference for high ceiling and open space is replicated in the current study but the preference for curvilinear contour is not. Even if a single-factor t-test [1] was employed for data analysis, the rating for curvilinear contours is still not significantly higher than the rating for rectilinear contours (P = 0.54 and 0.18 for beauty and pleasantness rating respectively). An important recent study has identified that experience can strongly modulate preference to curvilinear contours [18]. It is shown that, within the Western culture, experts, i.e., self-identified architects and designers, show stronger preference to curvilinear contours than non-experts. It is possible that increased experience or knowledge of Western architecture is associated with higher preference for curvilinear contours. ”*

18)More caution needs to be exerted when interpreting the results of this study. I find it confusing and misleading that the authors interpret the current findings as a cultural variation assessment in aesthetic preference of architectural features. This is not quite right, as the current study did not use participants from both Eastern and Western cultures so that to really compare the two cultures and to provide support for the cultural aesthetic variation claim.

However, the current findings can be linked to previous studies conducted in the Western culture, but the methodological differences between the current study and previous studies should be clearly stated (e.g., sample size, experimental procedure, statistical analyses used). This aspect should be considered in both discussion and abstract.

Thank you for pointing this out. We have now changed our claim and changed the title to *“Aesthetic Judgment of Architecture for Chinese Observers”*. Furthermore, we have mentioned clearly that the current study and the previous study on Western participants used the same stimuli and procedure, and we have also made it clear that there were potential differences across the studies, e.g., the sample size and statistics. Please also see the reply to the next question.

19)The authors should address the potential limitations of this study in a thorough manner, especially regarding the small sample size and statistical power.

Thank you for pointing this out. We have discussed potential limitations in *Discussion*:

*“Although the current study used the same stimuli and procedures as in reference [8] to facilitate the comparison between the two studies, there are some additional factors that may hinder such comparisons. For example, the two studies employed a different number of participants: The study by Vartanian.et al. 2013 recruited 18 participants and the current study recruited 29 participants. The detailed instructions are also presented in different languages and the data analysis procedures are not identical. To directly compare the architectural aesthetic preference across cultures, future studies need to recruit participants from different cultures, use carefully controlled instructions and ideally the same experimental environment, and analyze the data in the same way.”*

Reviewer #2: The manuscript presents an interesting study comparing the effects of contours, high ceilings and open spaces in interior environments on ratings of beauty and pleasantness. The study aims to replicate parts of a previous study (Vartanian, O., Navarrete, G., Chatterjee, A., Fich, L. B., Leder, H., Modroño, C., ... & Skov, M. (2013). Impact of contour on aesthetic judgments and approach-avoidance decisions in architecture. Proceedings of the National Academy of Sciences, 110(Supplement 2), 10446-10453.) but using Chinese participants rather than Western (Spanish) participants.

1)The title of the manuscript suggests the study examines cultural influence on the aesthetic judgement of architectural design. However, I think only very tentative conclusions can be drawn regarding culture. To make inferences about cultural influence, a direct comparison between participants from different cultures should have been carried out. This was not the case, only participants from an Eastern culture were used. Comparison against the results from an entirely different study (admittedly using the same stimuli, but no further details are given about the similarities between the two studies) can not provide conclusive evidence about cultural influences. Any differences between the two studies could have been due to a range of different reasons, other than culture.

Thank you for pointing this out. We have now changed our claim and changed the title to *“Aesthetic Judgment of Architecture for Chinese Observers”*. Furthermore, we have mentioned clearly that the current study and the previous study on Western participants used the same stimuli and procedure, e.g., the same presentation duration and the same rating conditoins. However, we acknowledge that we did not directly compare data for participants from different cultures and there were subtle differences across studies, e.g., the sample size and statistics. Please also see the reply to the question #3.

2)Another major issue I have with the manuscript is the lack of reflection on previous findings and how these relate to the findings reported in the study carried out by the authors. A large part of the Discussion section (e.g. lines 193-208) talks about potential differences between Western and Eastern perceptions of architecture, but there is virtually no discussion prior to this comparing the existing results with those found in previous literature, particularly in the study that this work is based on (Vartanian et al). I would like to see a more detailed description and comparative analysis carried out with the previous related literature. The authors state that “After comparing the results of this study with those of previous studies, we postulate that differences in viewers’ perceptions...is potentially related to cultural background” (lines 190192). However there is little to no evidence of this comparison of results.

Other issues for the authors to consider:

We have added comparions between the current results and the results in previous studies.

“*Previous studies have analyzed preference to architectural features in Western observers[1, 8, 18]. The current study and these previous studies employed the same 2 by 2 by 2 design to analyze how ceiling height, openness, and contour affect aesthetic rating, but the rating data were analyzed in different ways. Previous separately analyzed each factor while the current study employed a 3-way ANOVA analysis that considered the interaction between factors. Single factor analysis in these previous studies reliably showed that beauty and pleasantness rating is higher for space with higher ceilings than space with lower ceilings, higher for open space than enclosed space, and higher for curvilinear contours than rectilinear contours.* *The preference for high ceiling and open space is replicated in the current study but the preference for curvilinear contour is not. Even if a single-factor t-test [1] was employed for data analysis, the rating for curvilinear contours is still not significantly higher than the rating for rectilinear contours (P = 0.54 and 0.18 for beauty and pleasantness rating respectively). An important recent study has identified that experience can strongly modulate preference to curvilinear contours [18]. It is shown that, within the Western culture, experts, i.e., self-identified architects and designers, show stronger preference to curvilinear contours than non-experts. It is possible that increased experience or knowledge of Western architecture is associated with higher preference for curvilinear contours.* ”

3)A key limitation of the study that should be acknowledged is that the factors of interest couldn't be manipulated whilst keeping other variables constant. This should be acknowledged in the Discussion. It is possible that the factors investigated varied with some other unmeasured confounding variable/s.

We have discussed potential limitations in *Discussion*:

*“**Although the current study used the same stimuli and procedures as in reference [8] to facilitate the comparison between the two studies, there are some additional factors that may hinder such comparisons. For example, the two studies employed a different number of participants: The study by Vartanian.et al. 2013 recruited 18 participants and the current study recruited 29 participants. The detailed instructions are also presented in different languages and the data analysis procedures are not identical. To directly compare the architectural aesthetic preference across cultures, future studies need to recruit participants from different cultures, use carefully controlled instructions and ideally the same experimental environment, and analyze the data in the same way.* ”

4)Line 17 - reference [4] does not seem appropriate here. Some justification of sample size would be useful. Was no power analysis carried out based on the findings of the previous study (reference [5])?

The current study followed the same procedure used in the study by Vartanian.et al, and used the same stimuli. The study by Vartanian.et al recruited 18 participants and we recruited 29 participants. We have now mentioned that in Discussion

*“The study by Vartanian.et al. 2013 recruited 18 participants and the current study recruited 29 participants.”*

The pictures may have been from reference [5] (line 58), but were they classified in the same way (i.e. into the sub-groups if high/low ceiling, open/closed, and curvilinear/rectilinear? Please clarify. It is important to know whether you were using the same classifications as in the earlier study, or whether you carried out your own classification process.

We used the same classification. We have mentioned this in the manuscript.

Line 80 - reference missing.

We have added references.

5)It’s not clear why the total score for each set of images is used (giving a range of 25-125 for each image), rather than just take the mean score, which would be more intuitive as it would fit with the response scale used.

We have now used the mean instead of sum.

6)The authors state that simple effect comparisons do not need p-value adjustment (line 105), but p-values in the ANOVA do require adjustment.

We now only report the results for beauty rating in the main text, and therefore there is only 1 ANOVA, not requiring correction for multiple comparisons.

7)It is helpful that the raw data has been provided in the supplementary file, however this data needs some kind of codebook or readme file to explain how to understand and use it. Ideally the data for each participant would be put on one spreadsheet / csv file for ease of use, and definitions / explanations given for each variable. Furthermore, it would be useful to provide the analysis script as well, as a supplementary file.

All data and analysis scripts will be made online after the manuscript is accepted.

8)Lines 135-136 refer to pleasantness score, but should this be beauty score? The analysis of pleasantness appears to begin from line 138.

Participants rated both pleasantness and beauty. We have now clearly mentioned this:

*“Among the 4 combinations, compared to lower ceilings, higher ceilings were more likely to lead to a higher beauty score (p<0.05). The results for pleasantness rating were quantitatively similar to the results for beauty rating (Fig. S1) and were shown in Figs. S2 and S3.”*

9)The captions for Fig 2 and 4 include reference to 3 different digits, this is confusing and does not seem to match with the actual figures.

Thank you for pointing these out, we have changed the digits.

10)The footnote to tables 1 and 2 refer to effect size thresholds, and also that 90% CI is reported rather than 95% CI. Do you have references to support these statements?

We have changed it to the 95% CI.

11)S1 Figure shows the correlations between pleasantness and beauty. These are very strong - did you consider just combining the two ratings into one overall ‘aesthetic’ score? To analyse both separately seems unnecessary.

It is a great suggestion. However, since the study by Vartanian et al. separately analyzed these two factors and we aimed to compare our results with the results of Vartanian et al., we separately reported the two measures. We agree, however, that it is redundant to report both measures and therefore we have moved the results for pleasantness rating to Supplementary Materials.
