# Supplementary material for: Aesthetic judgment of architecture for Chinese observers
Source: PLoS One. 2022 Apr 5;17(4):e0265412. doi: 10.1371/journal.pone.0265412 (PMC8982842; doi:10.1371/journal.pone.0265412)
Supplement: Attachment — Submitted filename: Response to Reviewers.docx [file pone.0265412.s005.docx]

We would like to thank the reviewers for their constructive suggestions. Please see below for our point-to-point replies.

REVISIONS FOR THIS PAPER:

Introduction

- Citation needed at the end of this sentence - ‘Once a certain architectural element fits in a certain life scene, such as work, study, and rest, it can enhance behavioral effects through positive emotions.’

We have added references for the statements.

*“Once a certain architectural element fits in a certain life scene, such as work, study, and rest, it can enhance behavioral effects through positive emotions [7, 8] ”*

- I would suggest providing more evaluation and interpretation of the previous studies. For example, ‘it is suggested that the influence of openness on pleasantness and beauty involves in the anterior prefrontal cortex and the temporal pole.’ This is interesting, however, without mentioning some functional aspects, such as the role of the anterior temporal lobe in semantic memory and how that might relate to drawing meaning from our environment, the text is not convincing enough.

We have removed the discussion about neuroimaging results since the current study only analyzed behavior.

- In the last paragraph of the introduction, I would suggest clearly stating that this study was conducted in Chinese participants only. I find it confusing that the authors still use a comparison term with Western participants although they did not test Western participants.

We have clearly mentioned that the study only tested Chinese participants:

*“If the preference to architectural features is strongly influenced by daily architectural aesthetic experience, observers living in environments with different building styles may prefer different architectural features [20-22]. Here, we analyze the preference to architectural features, including ceiling height, openness, and contour, in Chinese observers.”*

We have now also clearly mentioned in the discussion that we do not compare our study with the previous studies.

*“**The current results suggest that Chinese observers prefer architectural space with high ceilings and open space. The preference to curvilinear contours interacts with ceiling heights and openness. The preference to high ceilings, open space, and curvilinear contours has also been shown for Western observers [1, 10, 22]. Since the current study only employs Chinese observers as the participant, it cannot quantify whether the preference to architectural features varies across cultures.”*

Methods

- Although the authors claim they did refer now to participants rather than to subjects, the term ‘subjects’ still appears 15 times in the method section.

We have made sure that “subjects” are now replaced with “participants”.

- ‘after completing the experiment each participant received a reward’ – Please provide more details about the nature of the reward received by participants.

We have added more details:

*“Each participant received 40 RMB monetary reward”*

- ‘Candidate participants who had knowledge about the design and process of this experiment were excluded’ – this is confusing, please mention how many participants were excluded from analyses.

The first and second authors were the first participants to test the experiment program. Since the authors knew previous studies and might have potential biases, their data were not analyzed. We agree that the statement is confusing and it is not necessary to report program testing. Therefore, the sentence is now removed.

- although the figure explaining the experimental procedure is very helpful, it is not clear whether the pictures were randomly presented or whether the order of different sets of pictures was fixed across participants.

The 200 pictures are presented in a randomized order for each participant. We have now mentioned:

*“The order of pictures was randomized for each participant.”*

- Citation needed when referring to Matlab and Psychtoolbox - ‘The experimental program was written using the MATLAB 2018 psychtoolbox software’.

A reference has been added.

Results
- although the authors have included a violin plot, it lacks important detail. It would be helpful if individual data were plotted out. At the same time, it is unclear which line represents 95% CI. Also, the text describing the conditions for the x-axis is very busy. I would suggest using a shortened name for conditions, background grid lines, individual data points plotted out and adding a legend explaining thoroughly the figure.

We have added individual data, and added more details to the figure and figure caption.

- In the current version of the manuscript, the effect size (partial eta squared) is missing. Please report the effect size.

Effect size has been added to Table 1 and Table 2.

- Are there any reasons for not including the results for pleasantness ratings in the main results section? - ‘The results for pleasantness rating were quantitatively similar to the results for beauty rating (Fig. S1) and were shown in Table. S1, Figs. S2 and S3’. I find it confusing for deciding to place those on the supplementary material, especially as in the discussion, the authors refer to both beauty and pleasantness rating results. Moreover, Vartanian et al., 2013 reported both pleasantness and beauty ratings in the main results section.

We have moved the pleasantness results back to the main results section.

Discussion

- The first and the last paragraphs in the discussion should make it unequivocally clear that this study was conducted with Chinese participants. As it stands, it is implying a Western comparison experimental condition, which is not true.

We have modified the result section:

*“The current results suggest that Chinese observers prefer architectural space with high ceilings and open space. The preference to curvilinear contours interacts with ceiling heights and openness. The preference to high ceilings, open space, and curvilinear contours has also been shown for Western observers [1, 10, 22]. Since the current study only employs Chinese observers as the participant, it cannot quantify whether the preference to architectural features varies across cultures.”*

- Paragraph two – citation needed after - ‘Preference for high ceilings and open space are consistent with previous results.’

The previous study refers to the study by Vartanian et al. Since a similar statement has already been made earlier, this sentence is now removed.

- I do not think it is useful to report p-values in the discussion section. I would suggest discussing how the results either support or do not support the hypotheses.

We have removed p-values in the discussion section.

Reviewer #2: I am grateful to the authors for their responses to my comments. The majority of my comments have been addressed adequately. However some responses do not fully address my original concerns.

Response to comment 3) - the additional discussion of limitation does not sufficiently mention the fact that the study was unable to change a factor of interest whilst keeping other factors constant. This introduces potential confounds and should therefore be mentioned clearly as a limitation. I do not think the authors’ current text does this.

We have thoroughly modified the manuscript to make sure that we no longer mention that we compare the results across studies. For example, in the discussion section, it is mentioned that

*“The current results suggest that Chinese observers prefer architectural space with high ceilings and open space. The preference to curvilinear contours interacts with ceiling heights and openness. The preference to high ceilings, open space, and curvilinear contours has also been shown for Western observers [1, 10, 22]. Since the current study only employs Chinese observers as the participant, it cannot quantify whether the preference to architectural features varies across cultures.”*

Response to comment 4) - just mentioning what the previous study’s sample size was is not the same as a power analysis. It would be useful to understand what effect sizes could have been detected with your sample size, and how these compared to the effect sizes that might be expected based on the previous Vartanian et al study.

We have now reported the effect size of the current study in Tables 1 and 2. We did not calculate the effect size for the Vartanian et al., study since the data are not publically available. None of the previous studies using the same picture set have reported the effect size, although the trend is consistent across studies [1] [10] [22] [29]. More importantly, we no longer compare our results with the Vartanian results.

Response to comment 5) - the text in the data analysis section still refers to the sum of the scores being calculated, with a value range of 25-125. This could be confusing, if you are now presenting mean values in the figures etc.

We have now consistently reported the mean values in figures and tables.

Response to comment 6) - the p-values in an ANOVA test still require adjustment to account for inflated Type I error. For example see this paper: Cramer, A. O., van Ravenzwaaij, D., Matzke, D., Steingroever, H., Wetzels, R., Grasman, R. P., ... & Wagenmakers, E. J. (2016). Hidden multiplicity in exploratory multiway ANOVA: Prevalence and remedies. Psychonomic bulletin & review, 23(2), 640-647.

We have now reported corrected P-value in Tables 1 and 2.

*“**The effect of multiple comparisons [25] were corrected using Bonferroni correction.”*

Response to comment 7) - it would be more helpful to include a better-described set of data, and analysis code, with the submission rather than after acceptance. This would ensure the data and its description / use is intrinsically part of the paper.

We have uploaded the data and code.
